# Proposal for a Standard Representation of Two-Dimensional Gel Electrophoresis Data

**DOI:** 10.1002/cfg.323

**Published:** 2003-10

**Authors:** Andrew Jones, Jonathan Wastling, Ela Hunt

**Affiliations:** 1 Department of Computing Science University of Glasgow 17 Lilybank Gardens Glasgow G12 8QQ UK; 2 Institute of Biomedical and Life Sciences University of Glasgow UK

## Abstract

The global analysis of proteins is now feasible due to improvements in techniques such as two-dimensional gel electrophoresis (2-DE), mass spectrometry, yeast two-hybrid systems and the development of bioinformatics applications. The experiments form the basis of proteomics, and present significant challenges in data analysis, storage and querying. We argue that a standard format for proteome data is required to enable the storage, exchange and subsequent re-analysis of large datasets. We describe the criteria that must be met for the development of a standard for proteomics. We have developed a model to represent data from 2-DE experiments, including difference gel electrophoresis along with image analysis and statistical analysis across multiple gels. This part of proteomics analysis is not represented in current proposals for proteomics standards. We are working with the Proteomics Standards Initiative to develop a model encompassing biological sample origin, experimental protocols, a number of separation techniques and mass spectrometry. The standard format will facilitate the development of central repositories of data, enabling results to be verified or re-analysed, and the correlation of results produced by different research groups using a variety of laboratory techniques.


**Comparative and Functional Genomics**
*Comp Funct Genom* 2003; **4**: 492–501.
Published online in Wiley InterScience (www.interscience.wiley.com). **DOI:** 10.1002/cfg.323

**Primary Research Paper**

### Proposal for a standard representation of two-dimensional gel electrophoresis data

Andrew Jones[1]*, Jonathan Wastling[2] and Ela Hunt[1]
[1] *Department of Computing Science, University of Glasgow, UK*
[2] *Institute of Biomedical and Life Sciences, University of Glasgow, UK*

**Correspondence to: Andrew Jones, Department of Computing Science, University of Glasgow, 17 Lilybank Gardens, Glasgow G12 8QQ, UK. E-mail: jonesa@dcs.gla.ac.uk*

### Abstract

**The global analysis of proteins is now feasible due to improvements in techniques such as two-dimensional gel electrophoresis (2-DE), mass spectrometry, yeast two-hybrid systems and the development of bioinformatics applications. The experiments form the basis of proteomics, and present significant challenges in data analysis, storage and querying. We argue that a standard format for proteome data is required to enable the storage, exchange and subsequent re-analysis of large datasets. We describe the criteria that must be met for the development of a standard for proteomics. We have developed a model to represent data from 2-DE experiments, including difference gel electrophoresis along with image analysis and statistical analysis across multiple gels. This part of proteomics analysis is not represented in current proposals for proteomics standards. We are working with the Proteomics Standards Initiative to develop a model encompassing biological sample origin, experimental protocols, a number of separation techniques and mass spectrometry. The standard format will facilitate the development of central repositories of data, enabling results to be verified or re-analysed, and the correlation of results produced by different research groups using a variety of laboratory techniques. Copyright © 2003 John Wiley & Sons, Ltd.**

**Keywords:** **proteomics; electrophoresis; standard; ontology**

### Introduction

Proteomics uses experimental techniques for the large-scale study of proteins. The experiments aim to determine all of the proteins expressed in a particular sample, or search for protein–protein interactions that may be crucial to the function of the system. Proteomics is a part of functional genomics, which also includes microarray analysis, phenotypic studies and small molecule arrays. The integration of all the diverse types of data is vital, and new bioinformatics tools are required (Tyers and Mann, 2003). In this work, we focus on the development of standards for studies of protein expression in which proteins are separated by two-dimensional gel electrophoresis (2-DE) and identified by mass spectrometry (MS). There is a major requirement for the development of a central proteome database that includes 2-DE images, analysis and MS data. For such a plan to be realized, it is vital that a standard data model is adopted by the research community to enable experiments from different laboratories to be compared or queried. A central database must contain sufficient detail about experimental protocols for the context of the experiment to be fully understood. It is also important that statistical analysis is captured, to ensure that new results derived from data are electronically accessible and can be verified. In other areas of biology, the deposition of data in a central repository is a prerequisite for publication: DNA sequences must be deposited in GenBank (**http://www.ncbi.nlm.nih.gov/Genbank/**) and protein structures in the PDB (**http://www.rcsb.org/pdb/**). Proteomics datasets can be very large,

Copyright © 2003 John Wiley & Sons, Ltd.

and not highly amenable to publication only in paper format, therefore a repository for published data would enable results to be obtained and understood more easily. A model representing the workflow of a proteomics experiment has been presented, known as PEDRo (Taylor *et al.*, 2003). The Proteomics Standards Initiative (PSI; **http://psidev.sourceforge.net/**) has been started by the Human Proteome Organisation (HUPO) and meetings have been held at the European Bioinformatics Institute (Orchard *et al.*, 2003a, 2003b). PSI is using PEDRo as an initial framework for the development of a standard. Our contribution is to develop a model for 2-DE data, difference gel electrophoresis, image analysis and statistical analysis of large datasets. We believe these data types are not adequately covered in the current proposals and offer our model as additional information that should be captured in the community standard. To justify this view, we focus on the proteomics techniques, the requirements of standards and the development of a data model.

### Proteomics techniques

A diagram describing the workflow of a proteomics experiment is displayed in Figure 1. Proteins are separated according to their charge and molecular weight using 2-DE, and are visualized by staining. The gel is scanned and analysis software detects properties of protein spots, including coordinates and an estimate of the volume of protein in the gel. Software can match spots produced on different gels corresponding to the same protein, and determine the relative difference in the spot size and intensity between two or more gels. Spot matching between gels is not perfect, therefore other techniques, such as MS, may be required

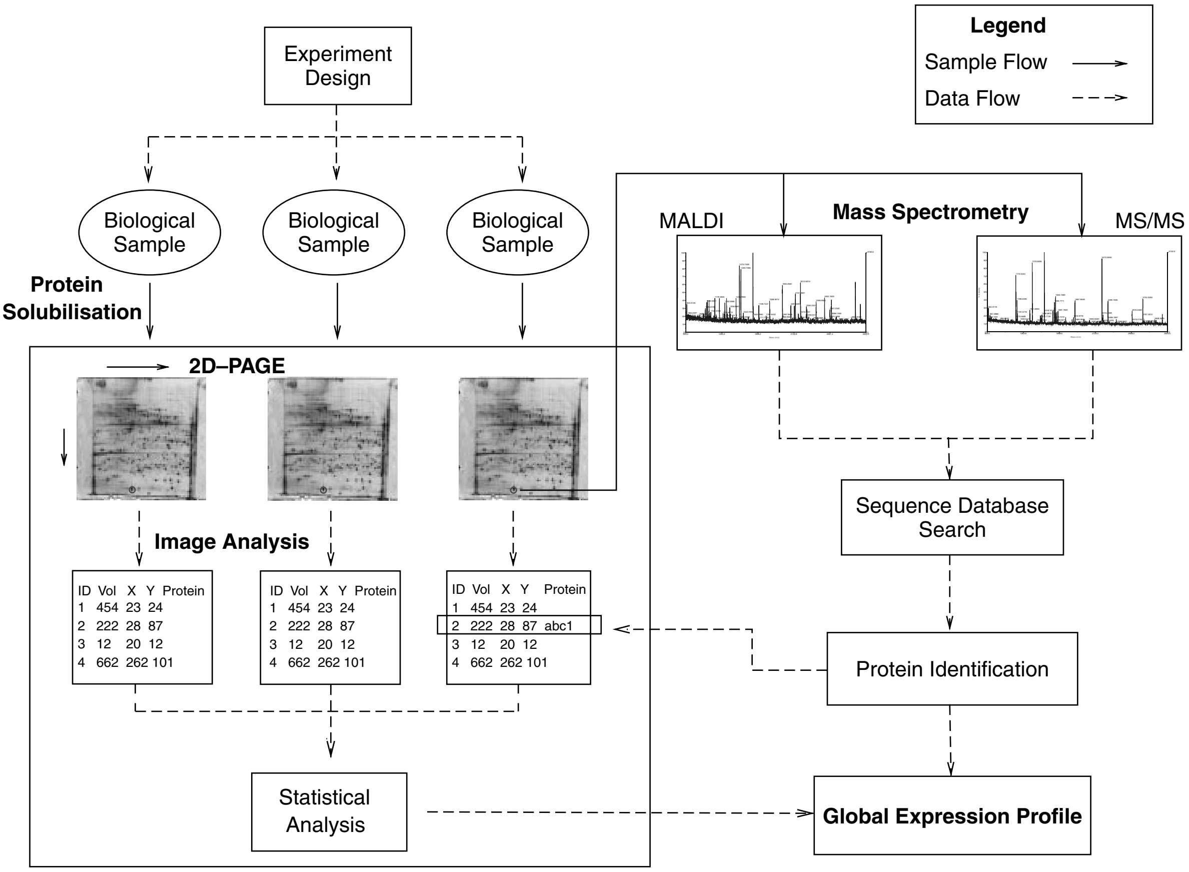

**Figure 1.** The data flow in a proteomics experiments. The area for which we have developed a model is boxed

Copyright © 2003 John Wiley & Sons, Ltd. 

to verify that matched spots belong to the same protein. In many cases, it is possible to determine the global expression profile of proteins in one or more samples of interest.

A new technology in protein separation is two-dimensional difference gel electrophoresis (Ünlü *et al.*, 1997) or DIGE (Ettan DIGE™ produced by Amersham Biosciences; **http://www.amershambiosciences.com**). DIGE enables two (or more) samples to be labelled with different fluorescent dyes, mixed and separated on a single gel. A proportion of the two samples can be mixed prior to gel loading and a third dye is applied, acting as an internal standard for calibration. The gel is scanned at different wavelengths, creating images that can be compared. This removes variability due to gel-specific differences and should greatly improve the detection of differential expression.

Protein spots may be excised from the gel, digested and analysed in a mass spectrometer. Information from an MS trace can be used to search sequence databases to determine the identity of a protein (a review of techniques is given by Mann *et al.*, 2001). After a protein has been identified there are a number of databases that may be accessed to characterize the protein. These include genomic or annotated sequence databases, repositories of protein motifs or families, and bibliographic references. It is important that the details of database searches are stored alongside experimental data to provide support for future re-evaluation.

### Requirement for standards

Proteome experiments are labour-intensive but can produce very large datasets which provide a summary of many proteins in a given sample. The development of robotic equipment, such as automated spot pickers, for high-throughput analysis, will further increase sizes of dataset. We have previously studied proteomics research activities in microbial pathogenesis and cancer research (Jones *et al.*, 2003). The studies highlighted several types of enquiry that benefit from proteomics techniques:

- *Proteome cataloguing*: determine the entire set of proteins expressed in a cell type, organelle or microorganism.
- *Hypothesis generation*: discover proteins whose function may be important in the condition of interest.
- *Protein regulation*: discover sets of proteins that share patterns of expression across a range of sample conditions.
- *Correlating gene and protein expression.*
- *Post-translational modifications*: identified by MS, which cannot be determined using microarrays.

The information from a proteomics experiment can provide an additional level of information to sequence databases (Aebersold and Mann, 2003). For example, if experiments to determine the proteome of human liver cells reveal that a specific protein is abundantly expressed, the information is functionally significant and should be available to researchers accessing sequence databases. Additionally, gel spots analysed by MS may reveal peptides that match a region of genomic sequence that has not been annotated. Therefore, the peptide sequences can be used to discover new genes, or edit incorrectly annotated genes.

The global protein profile generated by experiments depends upon the conditions under which the sample was produced and processed, prior to separation by 2-DE. The data may be valuable to researchers in diverse fields, who could obtain new results from datasets originally intended for another purpose. Therefore, it is vital that experimental protocols are rigorously documented according to a standard, and stored in a structured format that allows searches over biological conditions: species, cell, tissue type or experimental conditions, such as: gel constituents, stain, or MS instrument parameters.

### Model development

We have previously developed a prototype database for 2-DE and MS data (Jones, 2001) that highlighted challenges in data integration and capturing experimental protocol information in a structured format. We discovered that many types of questions cannot be answered using current technology, which would be solved by the development of a central repository and appropriate query tools. This motivated us to develop a model

Copyright © 2003 John Wiley & Sons, Ltd.

to describe data from 2-DE, difference gel electrophoresis, image analysis and statistical processing. The development of the model was driven by analysis of real datasets and an understanding of the types of queries that researchers would like to pose.

A repository for proteomics data must include a structured, detailed description of the origin of the sample. We believe that the standardization of sample origin should occur in conjunction with MGED (Microarray Gene Expression Data society; **http://www.mged.org**), who are developing microarray standards. There has already been significant work towards formal descriptions of sample origin which is highly applicable for proteomics.

Our model allows researchers to store data from any of the image analysis applications that are available. Statistical analyses performed on data produced from image analysis, such as software, algorithms and the associated parameters, can also be captured. The model is further specialized to manage difference gel electrophoresis data. Our model links spots visualized on a gel, to identified proteins, via MS. We do not include a proposal for annotation standards for MS; however, there are a number of groups working towards a standard for MS under the auspices of PSI, described in the next section. PSI will oversee the development of a complete model for proteomics that encompasses sample origin, 2-DE and MS. The complete model will provide the basis for a data exchange language and the schema for a central repository of data. The repository will provide the framework for integrating the diverse data types that encompass a proteomics experiment, and will enable researchers to query and analyse large datasets in ways that are not currently possible.

### An overview of previous work

### Microarray standards

A project was initiated for the standardization of microarray data formats, known as MIAME (Brazma *et al.*, 2001) (Minimum Information About a Microarray Experiment). MIAME defines all the data that must be stored about an experiment to allow it to be reproduced or analysed in light of other experiments. A formal specification of the requirements was released as an object model and a mark-up language called MicroArray and Gene Expression Markup Language (MAGE-ML) (Spellman *et al.*, 2002), in XML format (eXtensible Markup Language). The project is now managed by the MGED society. The work is highly relevant to the development of proteome standards for two reasons. First, there are a number similarities between microarray and proteomics experiments, therefore re-using aspects of the MAGE model will avoid repetition of work. Second, it is important that microarray and proteome data can be integrated, to enable co-analysis of gene and protein expression levels and determine the correlation between the two. The integration will be facilitated by similar representations of data.

MAGE captures details of the origin of a biological sample. This stage precedes the extraction of mRNA (microarrays) or proteins (2-D gels) and should be the same for both experiment types. There is a large variety of types of sample that could be used, therefore defining a model that allows all the information to be captured in a machine-readable format is a major challenge (Stoeckert and Parkinson, 2003). The starting sample could be cell culture, such as a mammalian cell line, therefore strain identifiers, growth media and timings must be captured. Experiments are also carried out on whole organisms, requiring the capture of genotypic and phenotypic information. The MGED group is developing an ontology of terms expressing how samples have been generated and the related parameters, e.g. the constituents of a bacterial growth medium. The function of the ontology is to provide the data types and values with which to populate the MAGE model. The controlled vocabulary is available from the MGED Ontologies working group (**http://mged.sourceforge.net/ontologies/**), and is used in the ArrayExpress database (**http://www.ebi.ac.uk/arrayexpress/**). It is likely that the MGED ontology will develop with contributors from different fields, including proteomics researchers adding terms relating to their own research area.

### PEDRo

PEDRo (Proteomics Experiment Data Repository; Taylor *et al.*, 2003) is a formal model to represent a proteomics workflow. PEDRo has been accepted

Copyright © 2003 John Wiley & Sons, Ltd. 

by PSI as a draft framework for the development of a standard. It consists of:

- Biological sample origin.
- Separation techniques, including 2-DE.
- Mass spectrometry laboratory protocols.
- Mass spectrometry data analysis.

PEDRo is designed to allow an experiment involving a number of stages of protein separation to be described, including 2-DE, affinity columns and chemical treatments. MS data is also described in the PEDRo model, including support for storage of database searches with results. There are a number of organizations developing standards for MS to serve different purposes (described below), therefore it is important that a consensus is reached.

The coverage of biological sample origin in PEDRo is focused on cell-based samples with limited coverage for whole organism proteomics. We believe that the work of the MGED society on sample origins should be incorporated into a proteomics standard. PEDRo covers many of the parameters needed to describe separation techniques; however, it has limited coverage of image analysis of single gels, and does not adequately capture multiple gel comparisons. We believe that PEDRo does not have sufficiently detailed classes to capture difference gel electrophoresis data completely.

### Proteomics databases

There are a number of databases accessible via the Internet which store proteomics data. SWISS-2DPAGE was initially developed in 1993, storing 2-DE images and information about proteins identified on gels with links to Swiss-Prot (**http://ca.expasy.org/sprot/**) and other databases. SWISS-2DPAGE has an interface with gel image maps that can be used to access information about protein spots (Hoogland *et al.*, 2000). Scripts are available for developing a database and interface based on SWISS-2DPAGE, known as make2ddb, which has been used to create a number of databases (links to the databases can be found at the SWISS-2DPAGE website: **http://ca.expasy.org/ch2d/**). SWISS-2DPAGE stores entries in a text format that includes bibliographic references, species of origin and pI/mW of proteins. However, the text format does not store substantial information about experimental protocols. The format stores peptide masses produced by MS, but without raw data or information about database searches that have been carried out, therefore the reliability of protein identification is difficult to determine.

A database has been developed by the Japanese Human Proteome Organisation (J-HUPO: **http://www.jhupo.org/**), which has an output format known as HUP-ML. HUP-ML is centred around 2-DE data and experimental protocols, allowing the constituents of solutions and timings to be specified, similar to sample preparation stages described in MAGE-ML. The format is likely to be mapped to the final format decided by PSI. There are a number of domain specific proteome databases, storing 2-DE or MS data (a summary of proteomics databases can be found at WORLD2D-PAGE: **http://us.expasy.org/ch2d/2d-index.html**). In general, the databases store only limited information about experimental protocols and are not fully integrated with other types of protein databases. It is a major challenge to integrate distributed proteomics databases, because data is not formatted in a uniform manner and the databases rarely offer wide-ranging query facilities.

### Mass spectrometry

Mass spectrometry is used in proteomics to identify proteins. An experiment generates raw data, in the form of a trace and processed data: a peak list corresponding to peptide masses. A major issue is coping with proprietary data formats generated by mass spectrometer manufacturers. Instruments are supplied with software for data collection and analysis. The software only provides the functionality to save analysis within a data format that cannot be interpreted by any other software. Researchers often manually enter the peak heights into a text editor, for input into database search programs. Proprietary formats pose a major problem for research throughput and data archiving. It cannot be assumed that the software needed to interpret the spectra will still be available in the future. It is also not feasible for researchers wishing to analyse the spectra deposited in databases to obtain the software that produced them. Therefore, there is a great need for a data exchange standard that can be interpreted without specialist software. The standard will support algorithm development for large scale database searches.

Copyright © 2003 John Wiley & Sons, Ltd.

There are several proposals for MS standards including GAML (Generalized Analytical Markup Language: **http://www.gaml.org**), and SpectroML (developed by the National Institute for Standards and Technology: **http://www.nist.gov**). GAML is an industry-generated effort to develop an XML-based data format for analytical instruments. GAML stores values of *x/y* coordinates from a trace, and the parameters entered in the instrument. SpectroML has similar goals and has been developed in collaboration with ASTM, an internationally recognized standards organization (**http://www.astm.org**). The PEDRo model also supports MS data. The goal of PSI is to unify the efforts, and work with instrument manufacturers to produce data formats conforming to the standard, once it is finalized.

### A model for 2-D gel electrophoresis and analysis

The data flow shown in Figure 1 outlines the stages in which information must be captured in a proteomics experiment. We have developed a model in UML (Unified Modeling Language: **http://www.uml.org/**) (Figure 2), to represent 2-DE data, image analysis and statistical processing for cross-gel analysis (see Figure 3). UML is a methodology designed to improve software engineering and requirements capture. UML class diagrams represent real-world objects or concepts using the definition of a *class* with certain *attributes* (see Figure 2). *Relationships* between classes can be expressed, representing the true relationships between concepts or datatypes. A UML model can subsequently be converted into a relational database schema or validation document for XML.

### Two-dimensional gel electrophoresis

A complex mixture of proteins can be separated by a number of techniques, including two-dimensional gel electrophoresis (2-DE), chromatography, affinity column and others. In this work we focus on 2-DE, which is the most widely used technique for protein separation in proteomics. A standard for 2-DE must capture the conditions under which the gel was run. The conditions include the dimensions and voltages applied to the pH strip, gel dimensions, buffers and staining procedures. Many of these parameters are covered in PEDRo, and we have reproduced certain attributes from PEDRo in the *2D-PAGE* class. Once a gel has been run, there is a significant amount of information that must be captured, which is not adequately covered in PEDRo. Initially, a gel is scanned and a raw image is produced. Our model incorporates this process, capturing the details of the scanner and the image produced, in the class *ScannedImage*. The model allows multiple instances of a scanning event in cases where researchers have re-scanned a gel, e.g. with scans at different resolutions. The image derived from a scanned gel becomes the input for the next part of the model — image analysis.

### Image analysis

A number of software packages can be used to analyse scanned 2-D gels. The software is able to perform edge detection on an image to determine

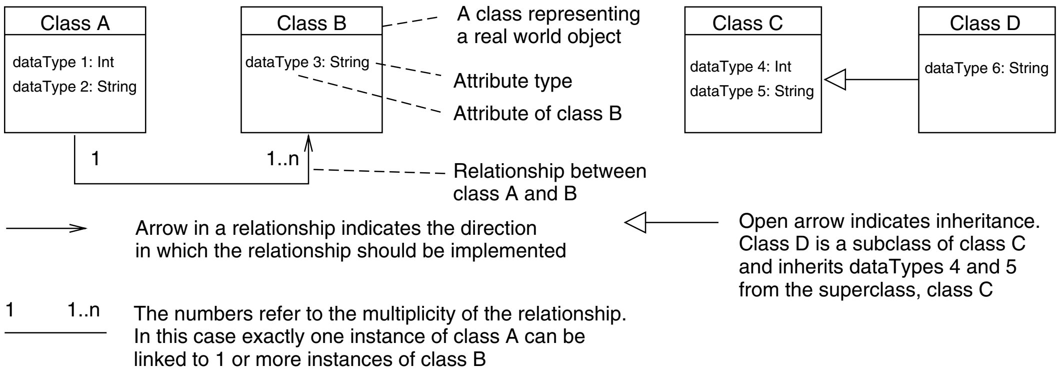

**Figure 2.** The main components of a UML class diagram

Copyright © 2003 John Wiley & Sons, Ltd. 

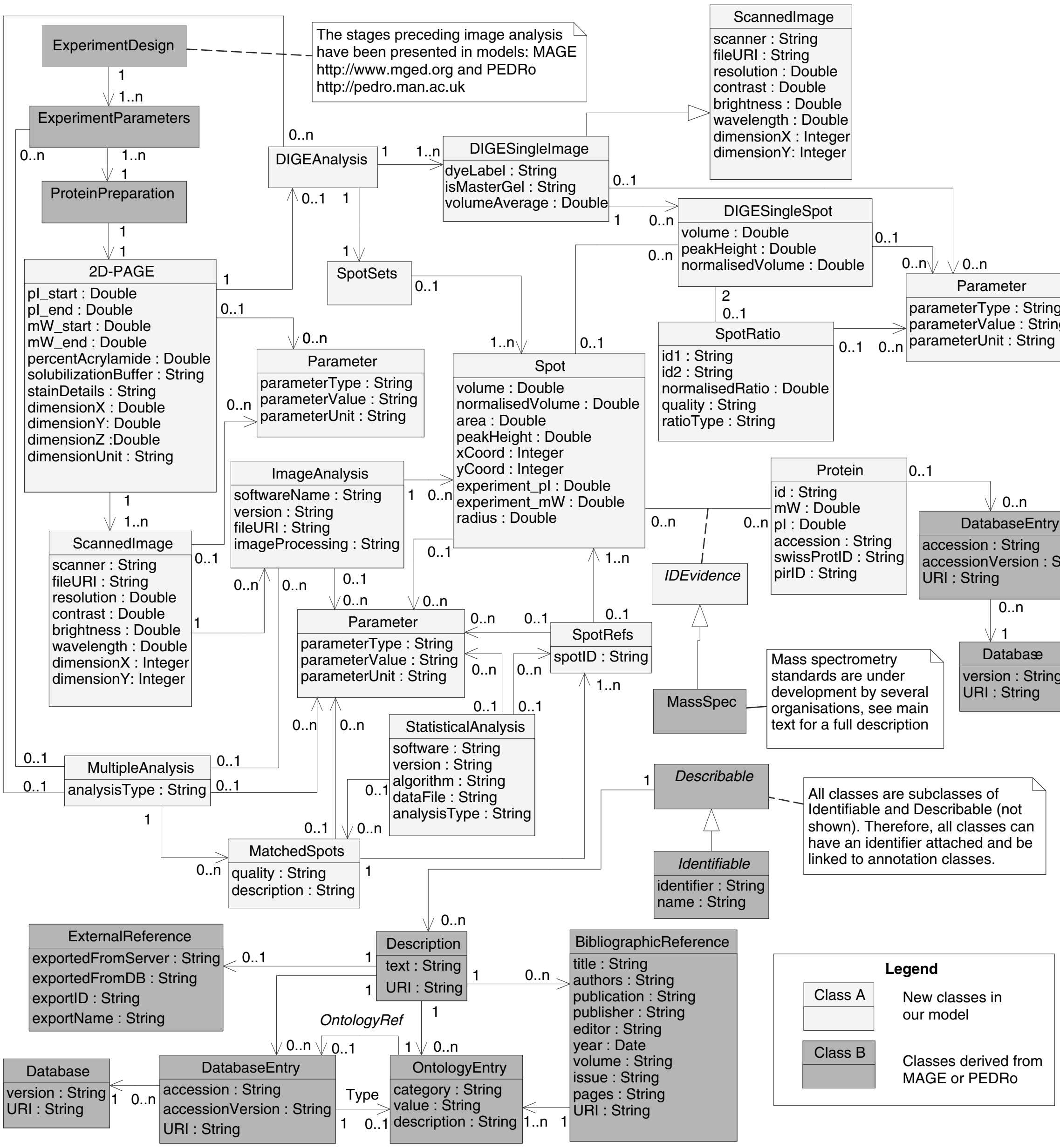

**Figure 3.** UML class diagram representing the relationships between types of data in an experiment using two-dimensional gel electrophoresis and mass spectrometry the coordinates, volume, area and other properties of protein spots. The class *Spot* accommodates many of the properties produced by current software packages; however, it is not possible to include all measures that may be produced by current or future software versions. A class containing the attributes *parameterName*, *parameterValue* and *parameterUnit* is used to cover datatypes that are not explicitly included in the model. Values for these attributes will be obtained from a controlled vocabulary to ensure consistency. The class *ImageAnalysis* records the software package and a description of image processing that has occurred.

Image analysis applications have the ability to match spots on different gels, believed to correspond to the same protein: from replicate gels for

Copyright © 2003 John Wiley & Sons, Ltd.

the same samples, or from gels over which a sample condition is varied, such as a time-course experiment. The overview of an experiment describes the number of replicates, with pointers to the protocol for the gel separation and the data. Spots that have been matched are linked via a specific class: *MatchedSpots* and the class *MultipleAnalysis* stores a description of the type of matching that has been carried out.

### Protein spots

There are separate classes for spots identified on a gel (*Spot*), and proteins (*Protein*) to which spots may be matched. The relationship between *Spot* and *Protein* allows one or more spot records to be linked to one or more protein records because there are known instances where a single spot in fact contains a number of different proteins. In the opposite direction, a protein record is likely to be matched to a number of spots on different gels, or possibly on a single gel. The relationship is linked via an attribute that includes the evidence for the match, such as any MS data that is available. This is vital because any findings based upon the predicted expression of a protein should take into account the probability that the protein has been identified correctly. The protein class contains sufficient information such that a repository based on the model can link directly to external databases. A single protein may have entries in a number of databases that may be relevant to the experiment, e.g. GenBank, Swiss-Prot, PIR (**http://pir.georgetown.edu/**) or domain-specific databases.

### Two-dimensional difference gel electrophoresis

Data produced from a difference gel electrophoresis experiment is described in our model. Amersham Biosciences produce DIGE technology and DeCyder™ software, for analysis of gels. DeCyder can export data in an XML format, known as DeCyderML (personal communication from Amersham Biosciences), which we have mapped to our model. A single gel produced using DIGE technology can produce several images, corresponding to the fluorescent dyes used for different samples. DeCyderML contains a class for the single channel image, with attributes such as dye type, which we have mapped to *DIGESingleImage*, which is a subclass of *ScannedImage*. A second class exists for single image spots (*DIGESingleSpot*). DeCyderML also has a class for storing information about co-migrated spots, which we have mapped to the general *Spot* class in our model. DeCyderML includes information about spots that have been matched across gels, which we have mapped to the *MultipleAnalysis* and *MatchedSpot* classes that exist in our model for storing non-DIGE data. DeCyder software calculates ratios between pairs of single image spots that have co-migrated, captured in *SpotRatio*.

### Statistical analysis

Statistical analysis techniques, such as ANOVA (analysis of variance), are used to locate spots whose volume is significantly different between two samples, indicating a change in protein expression under a certain condition. It is vital that the exact details of the analysis are preserved to ensure that the same procedure can be reproduced by other research groups. A number of statistical techniques can be applied to large datasets, such as analysis over a number of replicates, or over a number of gels analysing a varying condition. An example is cluster analysis, as performed on microarray data sets (Eisen *et al*., 1998), to detect groups of proteins sharing similar expression patterns over a number of gels. The *StatisticalAnalysis* class accommodates a description of the software or algorithm used to perform the analysis, and appropriate parameters and significance levels used. Our model has a link from a description of the analysis to the raw data. The analysis can be linked to individual spot records, or spots matched between gels. A formal description of statistical analysis presented by Papageorgiou *et al.* (2001) covers most of the attributes that are applicable to proteomics analysis, but is possibly too complex for use in biological applications. Our model has few attributes, with the intention that the details of the analysis will be described with data types obtained from controlled vocabularies. It is desirable that future versions of a proteomics standard incorporate future statistical standards.

### Annotation

We allow for annotation of all aspects of the experiment including raw data, experiment protocols and analysis. Annotation may be in the form of free text or links to external databases or ontologies.

Copyright © 2003 John Wiley & Sons, Ltd.

MAGE includes classes that allow annotation to be added and linked to any other part of the model. All classes in our model are subclasses of *Identifiable*, which allows an identifier to be added to each class. *Identifiable* is a subclass of *Describable*, which has a relationship to the annotation classes, therefore every class inherits this relationship.

### Discussion

### Web access to date

It has been recognized that past funding for large databases of scientific data has not been sufficient and, as a result, vital information is lost (Maurer *et al.*, 2000). An activity which attempts to remedy this situation is the effort to develop biochemical pathway databases, such as KEGG (**http://www.genome.ad.jp/kegg/**). Information regarding reaction kinetics and functional information has been published over several decades, but is not generally available in electronic form. Only papers published in the last decade may be available on the Internet, and data is not presented in any kind of format that can be mined automatically. Instead, information retrieval techniques must be used with significant manual intervention. This process is time consuming and will miss substantial amounts of information. In the current era, data regarding one biological system is often too extensive for a single researcher to gain access to by reading published literature, and automated methods are required. Microarray experts have previously recognized these needs and efforts are under way to develop large central repositories (Brazma *et al.*, 2000). The databases are intended to allow re-analysis of published data as new statistical techniques are developed. Microarray and proteomics experiments generate large amounts of data that is of potential use to researchers in many other fields. Improvements to algorithms for multiple analysis of 2-DE images will enable better use of repositories of gel images and will provide significant information about the mechanisms involved in protein regulation.

### Status of proteome standards

Our model represents data from one section of a proteomics workflow and complements other work undertaken by various organisations. PSI is overseeing the development of a standard, and is using PEDRo as an initial framework from which to develop a unified model. Our model covers image analysis of 2-DE, multiple gel comparison, DIGE and statistical analysis of large datasets, and represents additional information that we believe should be included in the next version of the community standard. Our model will be supplied to PSI, complementing PEDRo as the current proposal. We are currently integrating PEDRo with our model, to describe proteome-specific technologies, using MAGE components to capture experimental protocols. We believe that the MGED ontologies should be used to describe biological samples, and PSI should ensure close collaboration with MGED.

The development of a standard requires significant contribution from the proteomics community before consensus can be reached. The complete model should be flexible with regard to new technologies and experimental protocols. A data standard should not prescribe how researchers carry out experiments, but should capture enough detail to ensure that useful data archives can be developed. If a standard is to be accepted, it is vital that tools are developed that enable researchers to capture data conforming to the standard without substantial manual data entry. Laboratory Information Management Systems (LIMS) are available from commercial software vendors. They capture instrument parameters and track solutions using bar-coding. It is likely that future versions will be specifically tailored for proteomics applications, and software vendors should provide an output file conforming to the proposed standard. A dataset containing 2-DE images, MS traces, analysis and annotation is fairly bulky, therefore the development of a single, public database covering all aspects of proteomics is unlikely. A more feasible solution is the development of distributed, domain-specific proteome databases, such as single organism or disease databases. It is vital that databases provide wide ranging query facilities to enable the development of applications that search for data sets of interest. Data integration applications will be developed to link proteome databases to other repositories, such as databases of sequences, motifs and structures.

### Conclusions

We have developed a model to represent 2-DE image analysis, difference gel electrophoresis and

Copyright © 2003 John Wiley & Sons, Ltd.

statistical processing. Our model is a response to previous proposals for a standard for proteomics. PSI will oversee the development of a complete standard for proteomics. The standard will provide the basis for a central repository of data which will serve as a platform to integrate the diverse electronic resources that encompass a proteomics data set. The repository should facilitate re-evaluation of data and allow new data to be correlated with published results. We are currently developing a local repository for 2-DE and MS, with a schema developed from MAGE, PEDRo and our model. Our future work includes the development of XML storage technology to provide an integrated query system to a number of protein databases.

### Acknowledgements

The authors wish to thank Ashwin Kotwaliwale at the Beatson Institute for collaboration on the development of prototype databases. The development of the model was aided by discussions at the 2003 Proteomics Standards Initiative meeting at the European Bioinformatics Institute. Andrew Jones and Ela Hunt are supported by grants from the Medical Research Council. Jonathan Wastling's laboratory is funded by a grant from the BBSRC (17/513819).

### Related Websites

Amersham Biosciences; **http://www.amershambiosciences.com/**
ArrayExpress; **http://www.ebi.ac.uk/arrayexpress/**
ASTM International; **http://www.astm.org**
GAML; **http://www.gaml.org**
GenBank; **http://www.ncbi.nlm.nih.gov/Genbank/**
J-HUPO; **http://www.jhupo.org/**
KEGG; **http://www.genome.ad.jp/kegg/**
MGED; **http://www.mged.org**
MGED Network Ontology Working Group; **http://mged.sourceforge.net/ontologies/**
NIST; **http://www.nist.gov**
PDB; **http://www.rcsb.org/pdb/**
PEDRO; **http://pedro.man.ac.uk/**
PIR; **http://psidev.sourceforge.net/**
SWISS-2DPAGE; **http://ca.expasy.org/ch2d/**
Swiss-Prot; **http://ca.expasy.org/sprot/**
UML; **http://www.uml.org/**
WORLD2D-PAGE; **http://us.expasy.org/ch2d/2d-index.html**

### References

Aebersold R, Mann M. 2003. Mass spectrometry-based proteomics. *Nature* **422**: 198–207.

Brazma A, Hingamp P, Quackenbush J, *et al*. 2001. Minimum information about a microarray experiment (MIAME)-toward standards for microarray data. *Nature Genet* **29**: 365–71.

Brazma A, Robinson A, Cameron G, Ashburner M. 2000. One-stop shop for microarray data — Is a universal, public DNA-microarray database a realistic goal? *Nature* **403**: 699–700.

Eisen MB, Spellman PT, Brown PO, Botstein D. 1998. Cluster analysis and display of genome-wide expression patterns. *Proc Natl Acad Sci USA* **95**: 14 863–14 868.

Hoogland C, Sanchez JC, Tonella L, *et al*. 2000. The 1999 SWISS-2DPAGE database update. *Nucleic Acids Res* **28**: 286–288.

Jones A. 2001. A Database for Storing the Results of 2D-PAGE Experiments. Master's thesis, University of Glasgow. Contact author for a reprint at jonesa@dcs.gla.ac.uk

Jones A, Wastling J, Hunt E. 2003. Proposal for two-dimensional gel electrophoresis data standards. Tech. Rep. TR-2003-138, University of Glasgow. Contact author for a reprint at jonesa@dcs.gla.ac.uk

Mann M, Hendrickson RC, Pandey A. 2001. Analysis of Proteins and Proteomes by Mass Spectrometry. *Ann Rev Biochem* **70**: 437–473.

Maurer SM, Firestone RB, Scriver CR. 2000. Science's neglected legacy. *Nature* **405**: 117–120.

Orchard S, Kersey P, Hermjakob H, Apweiler R. 2003a. The HUPO Proteomics Standards Initiative meeting: towards common standards for exchanging proteomics data. *Comp Funct Genom* **4**: 16–19.

Orchard S, Kersey P, Zhu W, *et al*. 2003b. Progress in establishing common standards for exchanging proteomics data: the second meeting of the HUPO Proteomics Standards Initiative. *Comp Funct Genom* **4**: 203–206.

Papageorgiou H, Pentaris F, Theodoruou E, Vardaki M, Petrakos M. 2001. Modeling Statistical Metadata. In *Proceedings of the 13th International Conference on Scientific and Statistical Database Management*. IEEE Computer Society: Los Alamitos, CA; 25–35.

Spellman PT, Miller M, Stewart J, *et al*. 2002. Design and implementation of microarray gene expression markup language (MAGE-ML). *Genome Biol* **23**.

Stoeckert CJ, Parkinson H. 2003. The MGED ontology: a framework for describing functional genomics experiments. *Comp Funct Genom* **4**: 127–132.

Taylor CF, Paton NW, Garwood KL, *et al*. 2003. A systematic approach to modeling, capturing, and disseminating proteomics experimental data. *Nature Biotechnol* **21**: 247–254.

Tyers M, Mann M. 2003. From genomics to proteomics. *Nature* **422**: 193–197.

Ünlü M, Morgan ME, Minden JS. 1997. Difference gel electrophoresis: a single gel method for detecting changes in cell extracts. *Electrophoresis* **18**: 2071–2077.

Copyright © 2003 John Wiley & Sons, Ltd.
